# The main pulse of the Siberian Traps expanded in size and composition

**DOI:** 10.1038/s41598-019-54023-2

**Published:** 2019-12-10

**Authors:** L. E. Augland, V. V. Ryabov, V. A. Vernikovsky, S. Planke, A. G. Polozov, S. Callegaro, D. A. Jerram, H. H. Svensen

**Affiliations:** 1 Centre for Earth Evolution and Dynamics (CEED), University of Oslo, Oslo, Norway; 2 Sobolev Institute of Geology and Mineralogy Siberian Branch Russian Academy of Sciences, Novosibirsk, Russia; 3 Novosibirsk State University, Novosibirsk, Russia; 4 Trofimuk Institute of Petroleum Geology and Geophysics Siberian Branch Russian Academy of Sciences, Novosibirsk, Russia; 5 Institute of Geology of Ore Deposits, Petrography, Mineralogy and Geochemistry, Russian Academy of Sciences (IGEM RAS), Novosibirsk, Russia; 6 Volcanic Basin Petroleum Research (VBPR), Oslo Innovation Center, Oslo, Norway; 7 DougalEARTH Ltd, Solihull, UK

**Keywords:** Solid Earth sciences, Geochemistry, Geology

## Abstract

Emplacement of large volumes of (sub)volcanic rocks during the main pulse of the Siberian Traps occurred within <1 m.y., coinciding with the end-Permian mass extinction. Volcanics from outside the main Siberian Traps, e.g. Taimyr and West Siberia, have since long been correlated, but existing geochronological data cannot resolve at a precision better than ~5 m.y. whether (sub)volcanic activity in these areas actually occurred during the main pulse or later. We report the first high precision U-Pb zircon geochronology from two alkaline ultramafic-felsic layered intrusive complexes from Taimyr, showing synchronicity between these and the main Siberian Traps (sub)volcanic pulse, and the presence of a second Dinerian-Smithian pulse. This is the first documentation of felsic intrusive magmatism occurring during the main pulse, testifying to the Siberian Trap’s compositional diversity. Furthermore, the intrusions cut basal basalts of the Taimyr lava stratigraphy hence providing a minimum age of these basalts of 251.64 ± 0.11 Ma. Synchronicity of (sub)volcanic activity between Taimyr and the Siberian Traps imply that the total area of the Siberian Traps main pulse should include a ~300 000 km^2^ area north of Norilsk. The vast aerial extent of the (sub)volcanic activity during the Siberian Traps main pulse may explain the severe environmental consequences.

## Introduction

**Figure 1:**
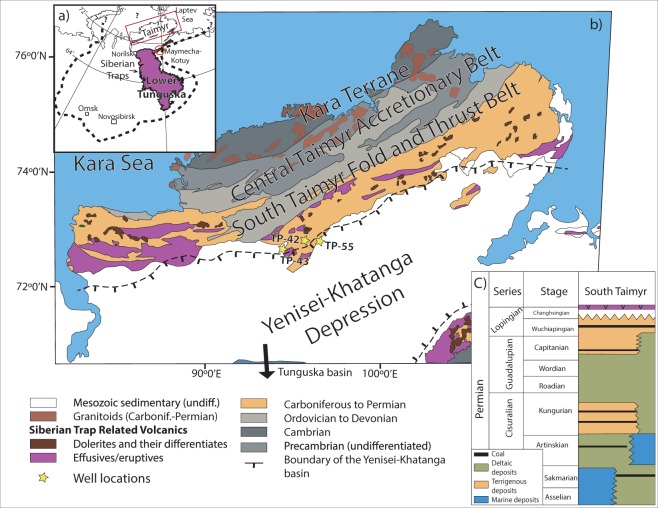
(**a**) Overview map of the Siberian Traps with the whole Siberian Traps province as proposed by Reichow *et al*.^10^ outlined by the dashed line. (**b**) Simplified geological map of the Taimyr Peninsula and the Yenisei-Khatanga Basin (modified from ref. ^74^), and (**c**) simplified and generalized stratigraphic column of Permian sedimentary rocks of South Taimyr (modified from ref. ^75^).

The Siberian Traps is one of the largest known large igneous provinces (LIPs) on Earth. It has been suggested to cover an area of up to 5 million km^2^ based on the extent of outcropping, seismically imaged and drilled basalts and dolerites in the Tunguska and West Siberia basins, north to the Taimyr Peninsula and into the Kara and Laptev Seas^1–14^. It is arguably one of the most important LIPs due to its potential role in the end-Permian extinction event at around 252Ma^2,15–22^. A key to understanding the impact of the Siberian Traps rests on the best constraints on the true volume of the event, the extent of subvolcanic intrusions, and on the key timing/duration of the main volcanic pulse (or pulses) that formed it. Synchronicity between the well-studied and well-dated main outcrop sequence of the Siberian Traps in the Tunguska basin^2,3,14,17,21^ and those in the surrounding areas was suggested by several workers^1,8,23–31^ and demonstrated at the ~5 m.y. level^10,11^, reflecting the uncertainty and accuracy in the geochronological data. However, this potential spread in ages is well outside the recently proposed time frame for the main pulse of Siberian Traps volcanic and subvolcanic activity at ca. 252.3–251.3 Ma^21^. Also, available data cannot resolve if subvolcanic and volcanic activity across the greater Siberia region (Fig. 1) was synchronous or not at the 0.1 Ma level, or if there are some specific geographical or compositional age trends.

The size and type of magmatic activity across a LIP is of great importance in understanding its formation and evolution, in particular when considering the environmental impacts of the subvolcanic and volcanic activity and the role of the sedimentary basins hosting the LIP^32^. One of the areas included in the greater region attributed to the Siberian LIP lies within the Taimyr Fold Belt on the Taimyr Peninsula (Fig. 1) and contains basaltic flows that have been stratigraphically, geochemically, and geochronologically (Ar-Ar), correlated to lava flows in the central part of the LIP^10,11,27,28,31^. It also contains numerous alkaline (ultra)mafic to felsic intrusive complexes in the form of layered intrusives, plutons, sills and dykes. Two of these alkaline layered intrusions, some doleritic sills and several smaller syenitic plutons have previously been dated to between ca. 249 to 230 Ma^33–36^, tentatively interpreted as a tailing stage of the Siberian LIP after the main pulse recorded in the Tunguska Basin between ca. 252.3 and 251.3 Ma^21^. However, as none of these rocks have been dated by high precision U-Pb geochronology, but rather Ar-Ar and U-Pb secondary ion mass spectrometry, age spread and uncertainties are large and the accuracy of the data is difficult to properly evaluate.

**Figure 2:**
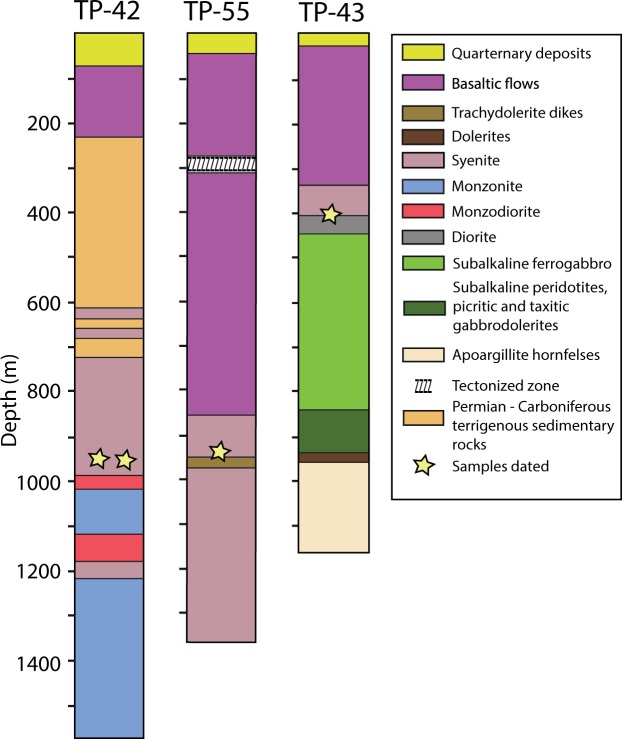
Lithological logs from the TP-42, TP-55 (Dikarabigai intrusive complex) and TP-43 drill cores (Dumtalei intrusive complex). The intrusive phases are emplaced into Permian- Carboniferous sedimentary rocks and flood basalts.

In order to test whether subvolcanic intrusives of more evolved compositions on the Taimyr Peninsula and in the vicinity of the Yenisei-Khatanga Trough are younger than the main pulse or can be related directly to it, we have sampled intrusions from boreholes from the Taimyr Peninsula for high precision U-Pb zircon geochronology. The samples come from different units within the Dumtalei and Dikarabigai ultramafic to felsic alkaline layered intrusive complexes (Figs. 1 and 2). As these intrusives cut the lower effusive volcanic pile at their locations, their ages may also provide a minimum age of the Taimyr volcanism. We, furthermore, investigate whether known alkaline rocks elsewhere in the Siberian Traps (i.e. in the Maymecha-Kotuy area) can be correlated with the Taimyr alkaline rocks based on geochemical data. In addition we evaluate the role of contact metamorphic devolatilisation, as the intrusions are emplaced within a sedimentary basin cf.^18^. An account of the geological setting of the Taimyr Peninsula and the Yenisei-Khatanga trough, as well as a summary of previous geochronology and other occurrences of alkaline magmatism potentially associated with the Siberian Traps can be found in Supplementary Material A.

## Results

### Petrology and geochemistry

**Figure 3:**
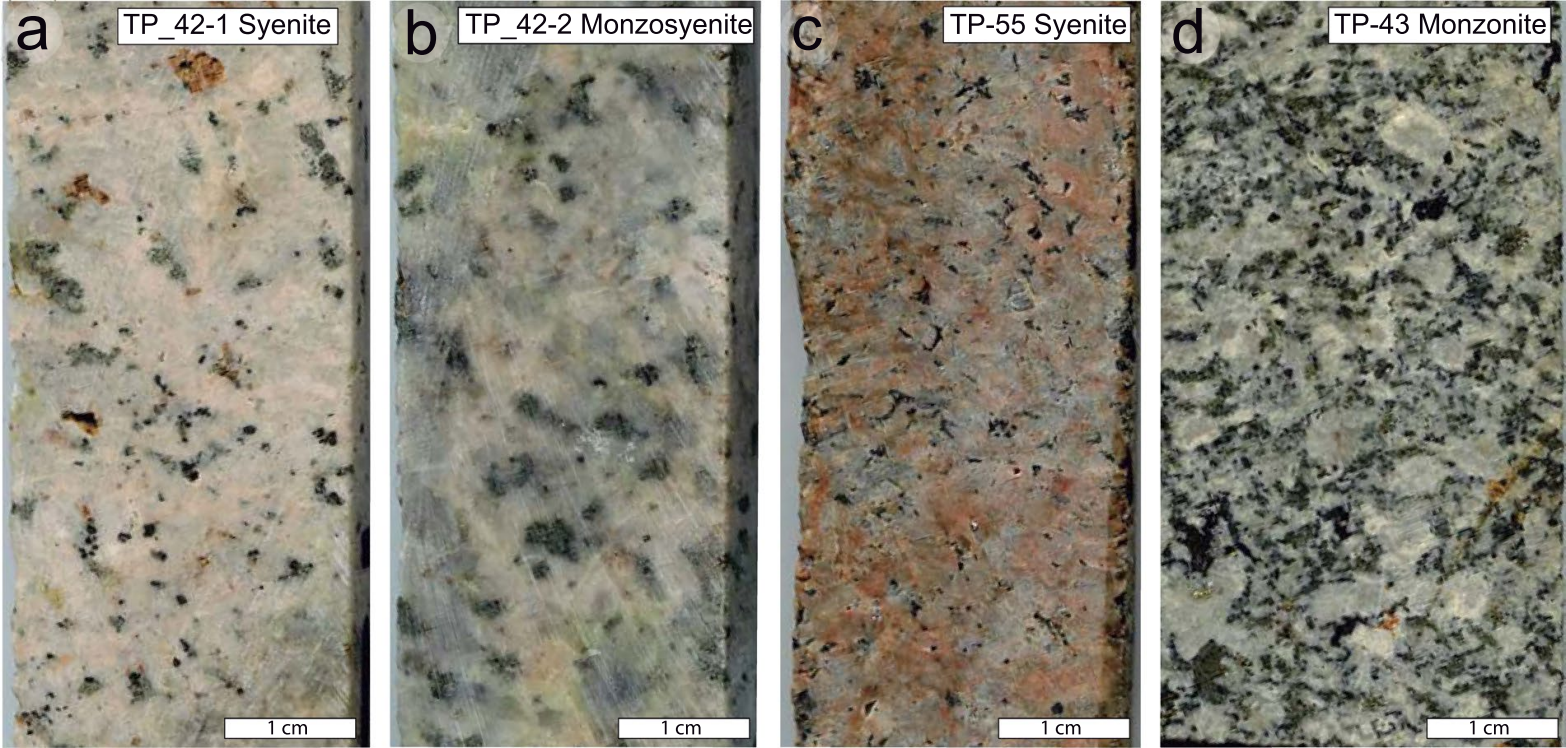
Scans of rock slabs from the sampled cores from the Dikarabigai intrusive complex (**a–c**) and the Dumtalei intrusive complex (**d**). (**a**) TP-42-1, (**b**) TP-42-2, (**c**) TP-55, (**d**) TP-43.

**Figure 4:**
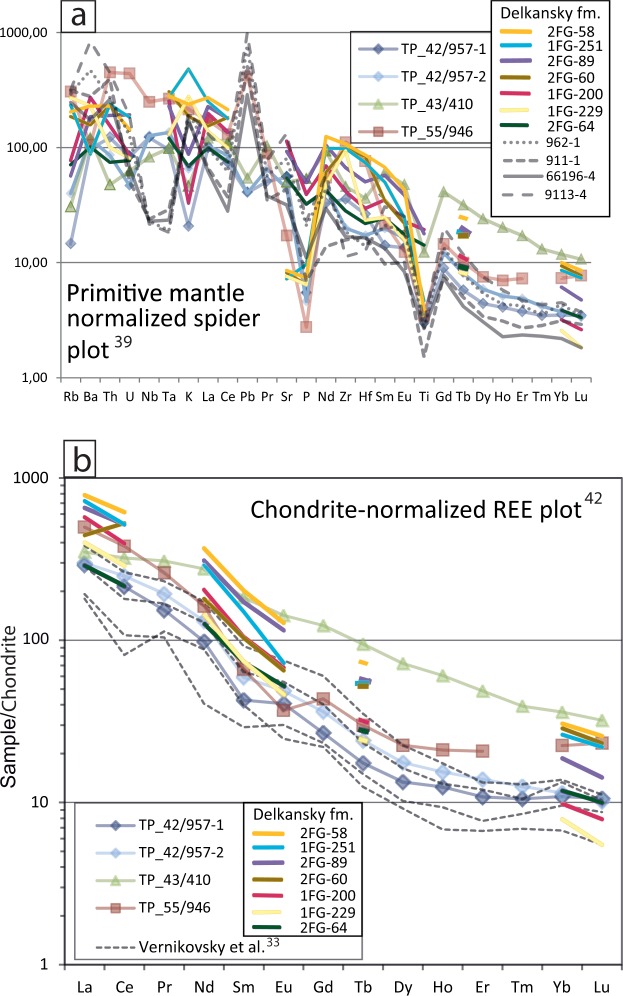
Trace element data from the samples analysed in this study (the Dikarabigai and Dumtalei intrusive complexes). Samples from syenitic plutons analysed by Vernikovsky *et al*.^33^ and volcanic rocks from the Delkansky formation of Meymecha-Kotuy area analysed by Fedorenko and Czamanske^38^ and Arndt *et al*.^37^ are plotted for comparison. (**a**) Spider diagram. (**b**) REE diagram. Delkansky formation samples 1FG-200, 2FG-64, 2FG-89 and 2FG-60 are from Arndt *et al*.^37^ and 1FG-251, 1FG-229 and 2FG-58 are from Fedorenko and Czamanske^38^. Samples 962-1. 911-1, 66196-4 and 9113-4 are from Vernikovsky *et al*.^33^ and represent synetic intrusives from western Taimyr.

Photos of rock slabs from four medium to coarse grained intrusive samples are presented in Fig. 3, ranging in composition from syenite (TP-42-1, TP-55), monzosyenite (TP-42-2) of the Dikarabigai intrusive complex to monzonite (TP-43) of the Dumtalei intrusive complex (Fig. 2; see Supplementary Material B for details of the setting and petrography of the Dumtalei and Dikarabigai intrusive complexes). There are modal and compositional variations within closely spaced samples (e.g. TP-42) as well as between intrusions themselves. The geochemistry of the analysed samples is presented in Supplementary Table S1 and Supplementary Fig. S1, along with additional reference to samples previously published by Vernikovsky *et al*.^33^, Arndt *et al*.^37^ and Fedorenko and Czamanske^38^. The incompatible trace elements (IE) of the three dated syenites are shown in Fig. 4a normalized to primitive mantle^39^. Zirconium values are the highest in syenite TP-55, and lowest in the monzosyenitic part of the TP-42 rocks. All four rocks share Ti negative anomalies, possibly reflecting early fractionation of Fe-Ti oxides. The monzonite sample TP-43 is the least enriched in the most IE and most enriched in the least IE, in accordance with its slightly more primitive character if compared with the syenites. The syenite TP-55 is the only rock showing a positive Pb anomaly, as opposed to the negative Pb anomalies shown by the other rocks. Potassium marks a negative through in all rocks except syeneite TP-55. Different amounts of fractionation and accumulation of K-feldspar can account for these differences. The pink colour of TP-55 (Fig. 3) is indeed indicative of K-feldspar accumulation in this sample. The three syenitic rocks show a negative P anomaly, not observed for the monzonite, probably reflecting early fractionation of apatite.

Previously, syenites and granosyenites from northern Taimyr were reported by Vernikovsky *et al*.^33^ to have characteristic features of A-type granitoids e.g.^40^. The IE of these rocks (Fig. 4a) show high Ba, Rb, Th and K, and a positive Pb spike, along with low Ta and Nb, which are typically retained as crustal signatures. Accordingly, Vernikovsky *et al*.^33^ reported that there was evidence of a considerable role for crustal assimilation, based also on high Ce and Sm abundances. Our samples follow quite well the IE patterns described by the syenites studied by Vernikovsky *et al*.^33^, except for the K positive anomaly and Nb-Ta negative anomaly, which are not observed in our samples.

Chondrite normalised^41^ REE data is plotted in Fig. 4b for comparison with Vernikovsky *et al*.^33^, Arndt *et al*.^37^ and Fedorenko and Czamanske^38^. The trends in REE pattern are remarkably similar for the syenite and monzosyenite samples, and deviate as expected for the more mafic monzonite (TP-43) (Fig. 4a). The lower REE abundances in the more evolved samples with respect to the least evolved one can be reconciled with the fractionation of REE-rich minerals (e.g. allanite) or phases in which REE can be less incompatible (e.g. amphiboles) during the evolution of these magmas. Notably, these phases were observed by Vernikovsky *et al*.^33^ in their samples. The negative Eu anomaly (0.69; calculated as Eu/Eu* = Eu/√(Sm × Gd)) shown by sample TP-55 again suggests an early fractionation of plagioclase from its precursor magma. Using these comparisons it seems plausible that the subalkaline examples from this study and those previously reported by Vernikovsky *et al*.^33^, share the same petro-genetic origin from a rather primitive mantle source, and a plausible crustal overprint. Estimates of the temperatures of the intrusions are relatively high in the range of 915–1080 (liquidus temperatures from whole rock data using the KWare MAGMA software; https://urldefense.proofpoint.com/v2/url?u=https-3A__www.lanl.gov_orgs_ees_geodynamics_Wohletz_KWare_Index.htm&d=DwIGaQ&c=vh6FgFnduejNhPPD0fl_yRaSfZy8CWbWnIf4XJhSqx8&r=W8RE-88OJ6HkLx5-vnSwGMbECZfnybKmwCXImBggWgpae5ASEwQeYcLKZnw2tz-i&m=31hZODtiHJvaUHOw_lUorTrBzoDxYvrGFlfGhKQPlsE&s=L7Og7cYLRwif3W_Tx4bKFg5IIocVbFBNpzgKr9Phboc&e).

### Geochronology

The mean ages reported below are presented with uncertainties as ±x/y/z, where x includes the analytical uncertainties, y includes the analytical and tracer calibration uncertainties and z includes the analytical uncertainties, tracer uncertainties and uncertainty in the decay constant.

**Figure 5:**
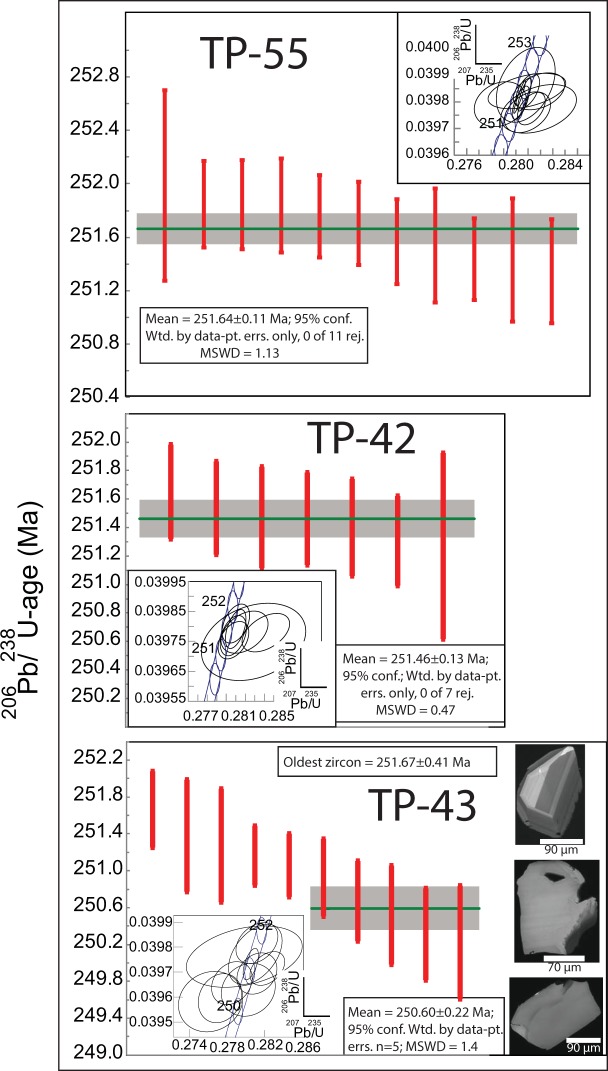
Geochronological data and representative Cl-images from zircons from sample TP-43 (lower panel). Abbreviations: Wtd.: weighted; pt.: point; errs.: errors; rej.: rejected.

Four high aspect ratio zircon prisms and seven zircon fragments from the syenite in drill core TP-55 were analysed by CA-ID-TIMS (Fig. 5; Table S2). The analyses are concordant to slightly discordant and overlapping, and give a weighted mean ^206^Pb/^238^U-age of 251.64 ± 0.11/0.16/0.31 Ma (2σ; MSWD = 1.13), which is considered to represent emplacement and crystallisation of the syenite. A total of seven single zircon fragments CA-ID-TIMS analyses from the syenite TP-42-1 and 2 are also equivalent within error (Fig. 3; Table S1), yielding a weighted mean ^206^Pb/^238^U-age of 251.46 ± 0.13/0.18/0.32 Ma (2σ; MSWD = 0.47). The two samples represent the medium and coarse grained phases of syenite layer, respectively and the calculated mean ^206^Pb/^238^U-age is considered to date the emplacement and crystallisation of the syenite layer. The age span recorded from the different phases of the Dikarabigai intrusive complex show that there was a prolonged evolution of emplacement and crystallization of the intrusive complex.

One euhedral, clear, slightly pinkish prismatic zircon, one euhedral, equant pyramidal and eight zircon fragments with some crystal faces were analysed by CA-ID-TIMS from the monzonite of drill core TP-43. All analyses are concordant but there is a significant spread in ^206^Pb/^238^U-ages (Fig. 5; Table S2). The oldest zircon gives a ^206^Pb/^238^U-age of 251.67 ± 0.41/0.43/0.51 Ma (2σ) interpreted to represent initial crystallisation in the magma system and probably emplacement of an early pulse of magma. The spread towards younger ages is interpreted to reflect a complex and long-lived crystallisation history of the layered intrusion and the five youngest equivalent (within uncertainties) zircons with a weighted average ^206^Pb/^238^U-age of 250.60 ± 0.22/0.25/0.36 Ma, are interpreted to represent the final emplacement and crystallisation of the monzonite-diorite horizon. The oldest and youngest zircons do not overlap in age supporting the interpretation of two (or more) magmatic crystallisation events (magma pulses) in the Dumtalei layered intrusive complex. Whether the older zircon ages represent antecrystic material from a long-lived magma chamber or xenocrysts from surrounding early phase magmatic rocks from the Dumtalei layered intrusive complex that was subsequently replenished by a second magma pulse is not immediately clear from the data. CL imaging reveals no visible xenocrystic cores in the CL-imaged grains (18 grains). There are, however, textural differences between the grains, ranging from magmatically zoned to faintly zoned to unzoned (Fig. 5). Some grains also show resorption textures, but the resorbed zones are generally minor (Fig. 5). This may indicate that older zircons were incorporated from a (partly) crystallised (mush) previous magma pulse during emplacement. This interpretation is supported by a rough estimate of the zircon saturation temperature based on the whole-rock composition and the zircon saturation model of ref. ^42^, giving a zircon saturation temperature (T_zirc_) of ca. 670 °C. Given the low T_zirc_, there should be no zircon antecrysts formed and preserved during the magma evolution. The most likely interpretation of the data is thus that the older zircons represent xenocrysts picked up from surrounding, previously emplaced, magmatic rocks of the Dumtalei intrusive complex, during emplacement. Alternatively, the spread towards younger ages could reflect late/post magmatic fluid assisted alteration/recrystallization of the zircons. There are, however, no indications of substantial alterations in thin section or in the zircons themselves, rendering such an interpretation unlikely. Nevertheless, the zircon data show that parts of the Dumtalei and Dikarabigai layered intrusions were emplaced synchronously within error and that final emplacement of the monzonite occurred at 250.60 ± 0.22 Ma.

## Discussion

**Figure 6:**
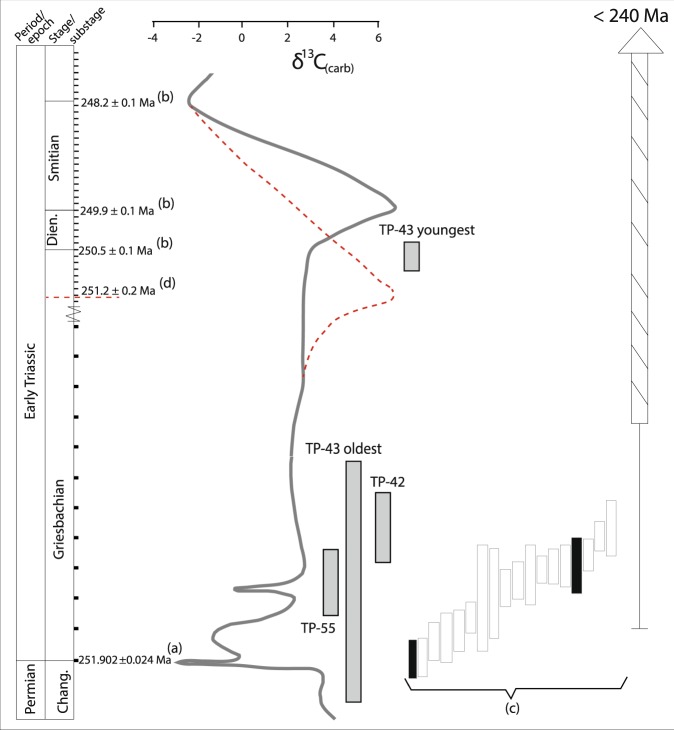
Earliest Triassic ages from Taimyr (this study) compared with ages of intrusive and effusive rocks of the main Siberian Traps. Carbonate carbon isotope curve from Meishan, China, with isotopic compositions from Cao *et al*.^76^, age calibrated by Burgess *et al*.^22^ and Li *et al*.^62^, is shown for reference. Note the change of scale in Ma before the onset of the Dinerian that is also reflected in the error bar of the age of TP-43. The alternative age calibration of the carbon isotope curve for the Dinerian and Smithian based on the Dinerian-Smithian boundary age of Galfetti *et al*.^60^ is shown as the red dashed curve for the Dinerian and Smithian stages. Weighted average ages for different phases of the layered intrusives presented in this study are represented by grey bars (2σ errors). The two ages for sample TP-43 are based on the youngest (n = 5) and oldest (n = 1) zircon analyses, respectively. The white and black bars (**c**) represent weighted average zircon U-Pb ages (2σ errors) from sills and effusives, respectively, from the Tunguska Basin and Meymecha- Kotuy presented in Burgess and Bowring^21^. Cross hatched bar represent the range of ages previously obtained from basalts, dolerites and layered intrusives from Taimyr with a 2σ error bar^10,11,34,35^. Stage boundary (Changshingian-Induan; (**a**)) is from Burgess *et al*.^22^, substage boundaries (**b**) are from Li *et al*.^62^, and alternative Dinerian-Smithian boundary marked by the horizontal red dashed line (**d**) is from Galfetti *et al*.^60^. For discussion on the uncertainty of the Early Triassic boundary ages see text.

This is the first high precision geochronological study from the Taimyr Peninsula that unequivocally shows that the alkaline (ultra)mafic to felsic layered intrusives are synchronous with the main pulse of volcanic and subvolcanic activity in the Siberian Traps^21^. The oldest dated syenite is slightly younger than the onset of the end-Permian mass extinction at 251.941 ± 0.037^22^ (not including tracer calibration uncertainties), but is identical to the average age of the main pulse of subvolcanic activity in the Tunguska basin^21^ (Figs. 5 and 6). This confirms previous suggestions e.g.^10,11,27,28,31^ that the main pulse of subvolcanic and volcanic activity of the Siberian LIP covered a significantly larger area than could be documented with existing data, but also show that it includes significant volumes of more chemically evolved magmas. The presence of alkaline intermediate to felsic rocks intruded during the main pulse of the Siberian LIP also calls into question the interpreted ages of the syenitic plutons dated by Vernikovsky *et al*.^33^, ranging from ca. 250–240 Ma. In fact the data used to calculate those ages show a lot of scatter and clearly display Pb-loss, rendering the assumption of concordance, and hence accuracy of the calculated ages, questionable. Moreover, their oldest dated sample at 249 ± 5 Ma, showing little sign of Pb loss, overlaps our data, an indication that at least some, if not all, of the other syenites in Taimyr and on islands in the Kara Sea were emplaced synchronously with the layered intrusives dated here and the main pulse of the Siberian Traps. This inference is supported by the geochemical overlap of the syenite samples studied here and those from the study of Vernikovsky *et al*.^33^, suggesting similarities in the petrogenesis between the samples (Figs. 4 and S1).

The intrusive nature of the alkaline layered intrusions into the lowermost volcanic pile combined with the new age data show that these volcanics were no younger than 251.64 ± 0.11 Ma and thus probably of the same age as the lower lavas from the Tunguska Basin. This confirms the previous suggestion by Reichow *et al*.^10,11^ based on Ar-Ar data that the Taimyr and Tunguska volcanics erupted synchronously. There are also indications in the geochronological data that the magmatic system feeding the Dumtalei layered intrusion was long-lived (Fig. 3). This could imply that there are also younger (<251 Ma) intrusives present within the Siberian Traps as has previously been indicated from a baddeleyite date from the Guli ultramafic-alkaline intrusive complex in the Maymecha-Kotuy area just south of the Yenisei-Khatanga Basin that yielded a weighted mean ^206^Pb/^238^U baddeleyite age of 250.2 ± 0.3 Ma^17^. The data are, however, discordant (the ^207^Pb/^206^Pb age is 278 ± 4) so potential residual Pb-loss, possibly giving a too young age, cannot be excluded; nevertheless it is overlapping with our youngest age indicated for the Dumtalei intrusion.

Tholeiitic magmatism dominates the Siberian Traps event in volume and areal extent. However, an important, localized phase of alkaline magmatism for this LIP was recognized in the Maymecha-Kotuy area, at the NE margin of the Siberian Traps province^37,43^. These magmas have been suggested to have a very deep mantle source and follow independent paths from the tholeiitic magmatism, reaching rapidly the surface without ponding in deep crustal magma chambers and escaping large interaction with the crust^36^. The latter authors put forward large volatile contents of the alkaline melts as possible explanation for this different behaviour, and this has been recently supported by Black *et al*.^19^ with data from volatile rich melt inclusions. In Maymecha-Kotuy, the Guli massif, a large ultramafic-alkaline intrusion^43–45^ is considered co-magmatic with some of the alkaline lavas^38^. We here compare data from our layered intrusive complexes from Taimyr, and syenite-granites of Vernikovsky *et al*.^33^, with the classical alkaline series in Maymecha-Kotuy.

For syenite–granite intrusive bodies in Taimyr Vernikovsky *et al*.^33^ invoked a hybrid mantle-crustal origin, on the basis of trace element geochemistry and Sr-Nd isotopes. We see a general overlap in trace element patterns between our rocks and those analysed by Vernikovsky *et al*.^33^ (2003), taking it as suggestive of similar origin for these evolved alkaline intrusive rocks. Isotopic data are not available for our samples, but given the similar provenance, petrography and major and trace element chemistry, net of some amount of crustal assimilation we can assign a similar origin to our rocks and those from Vernikovsky *et al*.^33^. It is important to note that any attempt of correlating our intrusive rocks with alkaline lavas on the basis of trace elements has to be considered cautiously. Indeed, as seen from the IE and REE patterns, the Taimyr intrusive rocks suffer of some accumulation/fractionation effect.

Fedorenko and Czamanske^38^ and Arndt *et al*.^37^ present a petrogenetic and volcanostratigraphic study on the tholeiitic (lower part) to alkaline (upper part) lava pile cropping out along the Maymecha River basin. They observe a large spread of geochemical compositions in the alkaline rocks compared to the tholeiitic sequence. The latter was described in the Noril’sk region by classical contributions e.g.^46,47^, defining 12 geochemically distinct lava units. Fedorenko and Czamanske^38^ correlate the lower part of the Maymecha with the main Noril’sk volcanic sequence, pointing out that the main tholeiitic series is stratigraphically lower than the alkaline one described in Maymecha. This is in agreement with the observation from the alkaline complexes cutting through lava piles in Taimyr. In the Maymecha Kotuy alkaline series, Delkansky is the only bimodal lava unit, showing rocks (trachydacites) with evolved compositions (SiO_2_ > 60 wt.%) comparable with those observed for our syenites and the syenites analysed by Vernikovsky *et al*.^33^. Also on the basis of REE concentrations and ratios comparisons, the Dikarabigai complex and the syenites described by Vernikovsky *et al*.^33^ can be correlated with the Delkansky formation as described by Fedorenko and Czamanske^38^ and Arndt *et al*.^37^. Interestingly, a trachyte tuff and a trachyrhyodacite tuff from the Delkansky formation were dated to 251.904 ± 0.061 Ma and 251.483 ± 0.088 Ma (not including tracer calibration uncertainties), respectively^21^ (black bars in Fig. 6). Burgess and Bowring^21^ suggested that there is a hiatus in the Delkansky formation between the two samples that are separated by ca. 135 m in the stratigraphy. Given the ages of Burgess and Bowring^21^ it is clear that the Taimyr intrusives were emplaced synchronously with the Delkansky formation and probably during the proposed hiatus in this lava stratigraphy.

Also the quartz-syenites and granite samples analysed by Kogarko *et al*.^45,48^ from the Guli complex are evolved. Major and trace element compositions for these rocks are not reported in the contribution, thus it is impossible to compare their REE and IE patterns with those of the Taimyr rocks and with the geochemical parameters defining the alkaline formations described by Arndt *et al*.^37^. However, on the basis of field relations, Kogarko and Zartman^45^ recognize six phases of magmatism building up the Guli Massif, and the evolved rocks are only present in a late phase (V out of VI phases, where the VI is the youngest), in agreement with the late (but not final) emplacement of the Delkanski lava suite in Maymecha Kotuy.

Radiogenic isotope data are available from the contributions of Arndt *et al*.^37^, Kogarko and Zartman^45^, Kogarko *et al*.^48^, Vernikovsky *et al*.^33^, and Malitch *et al*.^36^. Except from two outliers, the Sr-Nd isotope data show a growing crustal contribution from the juvenile component of ultramafic to mafic rocks from the Dumtalei layered intrusive^36^ (^87^Sr/^86^Sr_250Ma_ = 0.70454–0.70494 and ^143^Nd/^144^Nd_250Ma_ = 0.51250–51257) and the Delkansky rocks^37^ (^87^Sr/^86^Sr_250Ma_ = 0.7036–0.7046 and ^143^Nd/^144^Nd_251Ma_ = 0.5124–0.5125), to the Taimyr intrusions analysed by Vernikovski *et al*.^33^ (^87^Sr/^86^Sr_251Ma_ = 0.7047–0.7064; Nd/^144^Nd_251Ma_ ~ 0.5120), to the quartz-syenites and granites of the Guli complex^48^ (^87^Sr/^86^Sr_250Ma_ = 0.706–0.717 and ^143^Nd/^144^Nd_250Ma_ = 0.5116–0.5120). Interpreting these data, Arndt *et al*.^37^ suggest that the alkaline lavas were emplaced rapidly and virtually without interaction with the crust, Vernikovsky *et al*.^33^ recognize a hybrid mantle-crust origin for the Taimyr rocks, and Kogarko and Zartman^45^ call for a strong crustal contamination or even for an anatectic origin. As reported by Vernikovski *et al*.^33^, Taimyr intrusive bodies intruded the crust at shallow level. This is in agreement with fractionation paths calculated by Arndt *et al*.^37^, arguing for low pressure crystallization. Intrusive magma bodies are more prone to crustal assimilation and different contaminants or degrees of contamination might explain the differences in REE and IE patterns observed between the Dumtalei and the Dikarabigai layered intrusive complexes. For the Dumtalei intrusive complex this is supported by juvenile isotope signatures of the mafic components of the complex^36^.

The maximum areal extent of the Siberian Traps has been estimated to ca. 5 million km^2^, including Taimyr and the West Siberian basin where broadly coeval basalts and sills are present^9–12,27,28,31^ (Fig. 1). Our new data from Taimyr lend support to this estimate by documenting synchronicity between subvolcanic activity in Taimyr and the Tunguska Basin within a 100 ka uncertainty (Figs. 5 and 6). Although similar high precision data do not exist from the West Siberian basin, the results presented here show that the flood basalts in Taimyr were erupted prior to 251.64 ± 0.11 Ma and thus are not younger than those of the main Siberian Traps^21^. The fact that subvolcanic intrusives were emplaced at the same time as the main stage of volcanism in the Tunguska Basin (Fig. 6) corroborates a firm correlation with the main pulse of igneous activity in the Siberian Traps in its central part with that of the Taimyr. Hence, our data requires an upscaling of the volume of the main pulse of the Siberian Traps as previously suggested by e.g. Reichow *et al*.^10^.

In Taimyr, Permian coal bearing terrestrial sediments correlative with those in the Tunguska Basin, as well as early Paleozoic and older^49–54^ sedimentary rocks (shales, limestones, marls) are present. These probably have high organic carbon contents comparable to what has been documented from the Tunguska Basin^55^. Although not studied in the same detail, the general Neoproterozoic to Carboniferous stratigraphy of southern Taimyr, and the Yenisei-Khatanga basin, sitting between the Tunguska Basin and the Taimyr Peninsula, is very similar to that of the well-studied Tunguska basin. These areas to the north of Tunguska represented the passive continental margin to the Siberian platform from at least the upper Neoproterozoic to the early Carboniferous^49,51–53,56^.

Svensen *et al*.^18^ estimated that more than 100 000 Gt of CO_2_ could have been generated as a result of contact metamorphism of organic rich and petroleum bearing sediments around sill intrusions, based on an estimate of outcropping sill intrusions of 1.6 million km^2^ in the Tunguska basin. Assuming that the area containing the Taimyr traps and the Yenisei-Khatanga trough (Fig. 1) is also characterised by similar volumes of subvolcanic intrusions as the Tunguska Basin, this estimate may have to be scaled up by ca. 20%. The indications that the alkaline intrusions in Taimyr and the parent magmas to the Delkansky lavas were emplaced and assimilated and fractionated at a shallow crustal level in Permian organic rich sediments may actually have been an extra favourable situation for assimilation and mobilisation of organic carbon from the surrounding sediments, further adding to the carbon degassing budget.

Prolonged magmatism and CO_2_ degassing has been proposed as a mechanism for Early Triassic δ^13^C negative isotope excursions^57–59^. The indications in the geochronological data from the Dumtalei intrusive complex that magmatism was prolonged and that final emplacement of the studied monzodiorite occurred as late as at 250.60 ± 0.22 Ma shows that magmatic activity associated with the Siberian LIP continued into the Dinerian and possibly the Smithian. This assertion is also supported by the apparently overlapping ages of the Guli complex at the NE margin of the Siberian Platform^17^. The main uncertainty with this correlation is the yet unresolved ages for the Griensbachian-Dinerian and Dinerian-Smithian boundaries. Galfetti *et al*.^60^ reported a U-Pb zircon age for an ash bed just above the Dinerian-Smithian boundary of 251.22 ± 0.20 Ma, indicating that the age of the boundary was slightly older than our age. This age was in accordance with an existing ash bed age of 250.55 ± 0.51 Ma from the Early Spathian^61^. Li *et al*.^62^ on the other hand, recently estimated an age of 249.9 ± 0.1 Ma for the same boundary based on cyclostratigraphy tied to the precise Permian-Triassic boundary age of Burgess *et al*.^22^. However, this age estimate required large extrapolations from the nearest absolute anchor age, and its accuracy may be questionable. The presence of Dinerian to Smithian aged large intrusive complexes that intrude Paleozoic organic rich sedimentary rocks associated with the Siberian LIP and thus potentially causing thermogenic CO_2_ degassing, could provide an explanation for the δ^13^C negative isotope excursions^57–59^ observed in the Early Triassic. A possible relationship between intrusive activity and metamorphic carbon degassing associated with the Siberian LIP and the global environmental perturbations of e.g. the Smithian^58,59,63–66^ could be further explored through more high precision dating of intrusive complexes in Taimyr and elsewhere within the Siberian LIP if the uncertainties of the boundary ages in the Early Triassic are resolved.

## Samples and Methods

One sample from the TP-43 borehole of the Dumtalei layered intrusion (Fig. 2), monzonite sample TP43 (410 m below the surface, mbs; Fig. 3d), and two samples from the TP-42 borehole (Fig. 2), syenite sample TP-42-1 and monzosyenite TP-42-2 (957 mbs; Fig. 3a,b), and one sample from the TP-55 borehole (Fig. 2), syenite sample TP-55 (946 mbs; Fig. 3c), from the Dikarabigai intrusive complex were analysed for whole-rock major and trace element geochemistry (ICP MS/OES) and processed for zircon U-Pb chemical abrasion isotope dilution thermal ionization mass spectrometry (CA-ID-TIMS) geochronology.

### U-Pb geochronology

The samples were crushed, pulverised and reduced on a Wilfley table before separation of heavy minerals through standard magnetic and heavy liquid techniques at the University of Oslo. The zircons were selected under an optical microscope, annealed for ca. 72 hours at ca. 900 °C and chemically abraded with HF (+HNO_3_) at ca. 195 °C for 14 hours^67,68^. The zircons grains chosen for analyses were spiked with a mixed ^202^Pb-^205^Pb-^235^U tracer that has been calibrated to the EARTHTIME (ET) 100 Ma solution^69^ by measurement with the exact same instrument procedures as the unknown samples (giving a weighted mean age of 100.209 ± 0.038 Ma; 2σ, MSWD = 0.72, n = 12). This intercalibration allows direct comparison of dates generated with our in-house tracer with dates generated by the ET tracers (making sure that tracer calibration uncertainties are included in the ages when compared). After spiking, the zircons were dissolved in HF (+HNO_3_) at ca. 210 °C for >48 hrs in Teflon micro capsules enclosed in a Parr type Teflon bomb. The solutions were subsequently chemically separated through column chemistry (separating U and Pb from REE’s and other ionisation inhibiting elements). The solutions were loaded on zone refined Re filaments and measured on a Finnigan MAT262 thermal ionisation mass spectrometer (TIMS). For all samples Pb was measured in dynamic mode on a Masscom secondary electron multiplier and U in static mode on Faraday cups. Corrections for Pb fractionation were made using the measured ^202^Pb/^205^Pb-ratios for each Pb analysis relative to the ^202^Pb/^205^Pb-ratio of the tracer of 2.2702 (±0.006%, 2σ). The long-time average U-fractionation determined from measurements of the U500 standard solution (0.07%/a.m.u. ± 0.04%, 2σ) was used to correct for sample U-fractionation. Pb blanks are generally ≤1 pg (with composition: ^206^Pb/^204^Pb = 18.04 ± 0.40%; ^207^Pb/^204^Pb = 15.22 ± 0.30%; ^208^Pb/^204^Pb = 35.67 ± 0.50; 1σ), and in general all common Pb is considered to represent Pb blank, also for analyses no. 4 and 6 from TP-43 where additional common Pb was present. The raw data were reduced using Tripoli^70^ and analytical errors and corrections (including tracer uncertainties and Th-corrections, assuming Th/U in the magma of 3) were incorporated and propagated using an Excel macro based on published algorithms^71^. Weighted mean dates were calculated using ISOPLOT^72^ with specified decay constants^73^ and are presented in Table S2.

## Supplementary information

Supplementary Information
