# Supplementary material for: The main pulse of the Siberian Traps expanded in size and composition
Source: Sci Rep. 2019 Dec 10;9:18723. doi: 10.1038/s41598-019-54023-2 (PMC6904769; doi:10.1038/s41598-019-54023-2)
Supplement: Supplementary file 1 — Supplementary Information [file 41598_2019_54023_MOESM1_ESM.pdf]

Supplementary material

Manifestation of the Siberian Traps in Taimyr, Arctic Siberia: high precision U-Pb geochronology of alkaline layered intrusive complexes

L. E. Augland, V.V. Ryabov, V.A. Vernikovsky, S. Planke, A. Polozov, S. Callegaro, D. A. Jerram and H. H. Svensen

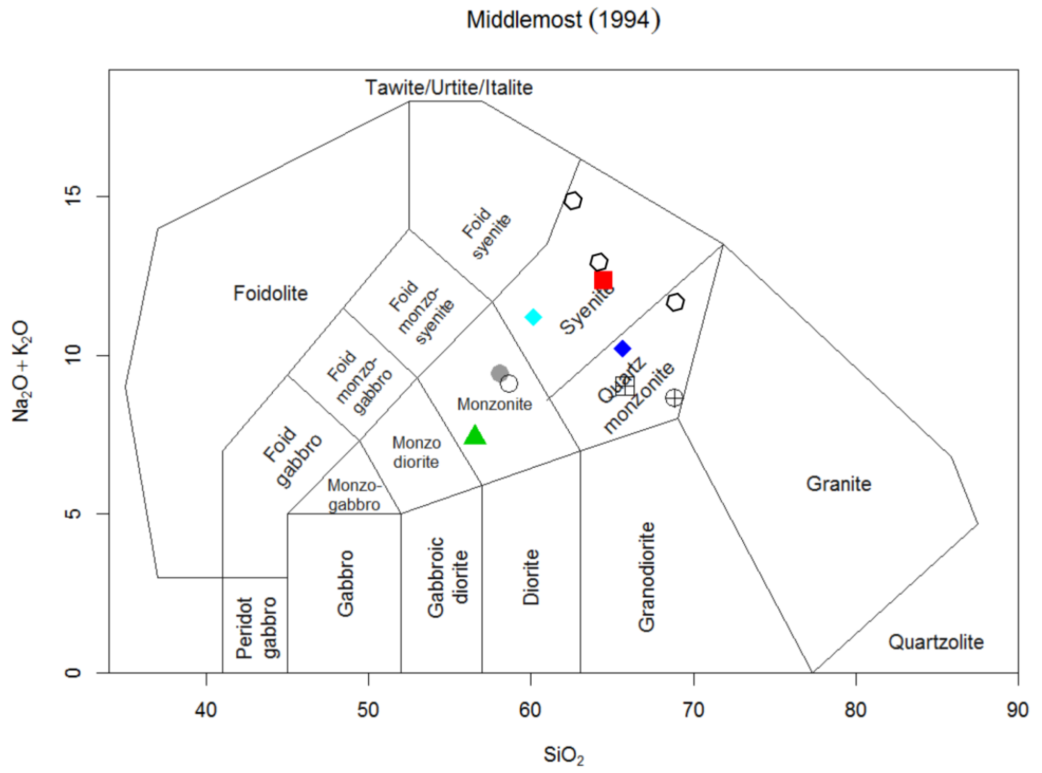

Figure S1: TAS diagram[51]. Deep blue and sky blue diamonds are TP_42/957-1 and TP_42/957-2 quartz monzonite and syenite, respectively; green triangle is TP_43/410 monzonite; red square is TP_55/946 syenite (this study). Grey and empty circles are 962-1 and 9113-4 monzonites, respectively; and crossed circle and square are 911-1 and 66196-4 quartz monzonites, respectively (from ref. 30). Open hexagons are extrusives from the Delkansky formation in the Maymecha-Kotuy area (from ref. 46).

Table S1: Geochemical data.

Sample	TP_42-1	TP_42-2	TP_43	TP_55
SiO2	64.16	56.38	55.48	63.0
TiO2	0.65	0.78	2.66	0.75
Al2O3	18.72	18.44	14.79	18.6
Fe2O3T	0.81	1.02	6.48	1.21
MnO	0.05	0.03	0.34	0.1
MgO	0.42	0.43	3.79	0.58
CaO	2.9	6.12	6.83	1.51
Na2O	9.35	8.55	5.88	5.62
K2O	0.63	1.96	1.39	6.47
P2O5	0.12	0.1	1.17	0.06
Rb	9.29	25.24	19.40	194
Cs	0.15	0.35	0.16	1.12
Sr	1174.8	931.63	1060.17	363
Ba	690.39	830.56	849.03	738
Sc	0.68	0.79	8.71	0.76
V	9.55	12.34	100.16	8.2
Cr	3.73	3.03	3.62	2.58
Co	1.51	11.03	11.27	1.15
Ni	0.08	2.66	2.64	0.09
Cu	4.62	5.73	13.13	4.8
Zn	27.39	16.38	116.3	43.52
Ga	21.92	17.39	26.76	26.71
Y	18.7	23.44	86.24	33.89
Zr	404.59	223.09	514.06	1244
Nb	87.96	90.59	58.94	178
La	68.97	70.83	82.9	118
Ce	131.35	152.43	196.98	233
Pr	14.21	17.93	28.57	24.1
Nd	44.97	60.05	125.75	74.1
Sm	6.33	8.76	27.06	9.82
Eu	2.29	2.74	8	2.08
Gd	5.34	7.23	24.51	8.66
Tb	0.63	0.87	3.42	1.08
Dy	3.29	4.33	17.7	5.54
Ho	0.68	0.84	3.31	1.15
Er	1.73	2.22	7.79	3.31
Tm	0.26	0.31	0.97	1.26
Yb	1.75	1.84	5.81	3.61
Lu	0.26	0.25	0.79	0.57
Hf	8.26	5.33	11.21	23.53
Ta	3.99	5.55	4.03	10.9
Pb	2.9	2.85	3.84	29.79
Th	10.48	7.3	4.03	38.21
U	1.77	0.98	1.31	9.24

Table S2: U-Th-Pb isotopic data

Compositional Parameters				Isotopic Ratios							Isotopic Ages					
Sample	Th/U	Pb*/Pb_c	Pb_c (pg)	$^{207}Pb/^{206}Pb$	% err	$^{207}Pb/^{235}U$	% err	$^{206}Pb/^{238}U$	% err	corr. coef.	$^{207}Pb/^{206}Pb$	±	$^{207}Pb/^{235}U$	±	$^{206}Pb/^{238}U$	±
(a)	(b)	(c)	(c)	(e)	(f)	(e)	(f)	(e)	(f)		(g)	(f)	(g)	(f)	(g)	(f)
**TP-43**																
1	0.74	22	1.2	0.05115	0.57	0.2808	0.60	0.039812	0.17	0.334	248	13	251.3	1.3	251.67	0.41
2	0.80	19	1.2	0.05116	0.70	0.2805	0.78	0.039766	0.25	0.463	248	16	251.1	1.7	251.38	0.61
3	1.4	8	0.71	0.0508	1.6	0.279	1.6	0.039750	0.25	0.294	233	36	249.5	3.6	251.28	0.62
4	1.0	19	1.1	0.05155	0.50	0.2823	0.53	0.039714	0.14	0.392	266	11	252.5	1.2	251.06	0.34
5	1.1	52	0.68	0.05121	0.19	0.2805	0.24	0.039732	0.13	0.579	250.1	4.5	251.07	0.53	251.17	0.32
6	0.94	25	3.7	0.05145	0.63	0.2816	0.65	0.039696	0.17	0.238	261	14	251.9	1.4	250.95	0.42
7	0.89	32	1.3	0.05089	0.60	0.2782	0.61	0.039653	0.18	0.231	236	14	249.3	1.4	250.68	0.43
8	0.76	40	2.0	0.05126	0.83	0.2801	0.83	0.039627	0.22	0.138	252	19	250.7	1.8	250.52	0.54
9	1.1	69	1.3	0.05076	0.68	0.2771	0.68	0.039594	0.20	0.169	230	16	248.4	1.5	250.32	0.50
10	0.76	42	1.4	0.05058	1.0	0.276	1.0	0.039580	0.25	0.118	222	23	247.5	2.2	250.23	0.61
**TP-42-1**																
1	1.6	49	0.46	0.05149	0.59	0.2824	0.63	0.039775	0.14	0.382	263	14	252.5	1.4	251.44	0.35
2	1.4	19	0.94	0.05188	0.54	0.2845	0.59	0.039772	0.13	0.449	280	12	254.2	1.3	251.42	0.32
3	2.0	17	0.87	0.05163	1.43	0.2829	1.47	0.039738	0.26	0.251	269	33	252.9	3.3	251.21	0.65
4	2.4	13	0.78	0.05136	0.2	0.282	0.3	0.039789	0.13	0.550	257.1	5.4	252.06	0.63	251.52	0.33
**TP-42-2**																
1	1.8	1	1.3	0.0497	0.6	0.272	0.6	0.039776	0.20	0.293	180	13	245	1	251.4	0.5
2	2.7	40	0.83	0.051320	0.34	0.2817	0.37	0.039806	0.13	0.437	255.2	7.7	251.98	0.83	251.63	0.33
3	2.2	79	1.2	0.051335	0.33	0.2815	0.36	0.039766	0.14	0.407	256	8	251.8	0.8	251.38	0.34
4	2.2	45	0.77	0.051329	0.36	0.2813	0.39	0.039750	0.13	0.424	255.6	8.2	251.70	0.87	251.28	0.31
5	1.8	26	0.98	0.050627	5.26	0.2780	5.48	0.039830	0.35	0.653	223.9	122	249.1	12.1	251.78	0.88

Table S2 cont.: U-Th-Pb isotopic data

Compositional Parameters				Isotopic Ratios							Isotopic Ages					
Sample	Th/U	Pb^*/Pb_c	Pb_c (pg)	$^{207}Pb/^{206}Pb$	% err	$^{207}Pb/^{235}U$	% err	$^{206}Pb/^{238}U$	% err	corr. coef.	$^{207}Pb/^{206}Pb$	±	$^{207}Pb/^{235}U$	±	$^{206}Pb/^{238}U$	±
(a)	(b)	(c)	(c)	(e)	(f)	(e)	(f)	(e)	(f)		(g)	(f)	(g)	(f)	(g)	(f)
**TP-55**																
1	1.4	69	0.81	0.051243	0.64	0.2816	0.69	0.039863	0.29	0.380	252	15	252.0	1.5	251.98	0.71
2	0.9	51	0.61	0.051296	0.21	0.2818	0.26	0.039840	0.13	0.556	254.2	4.9	252.06	0.57	251.84	0.32
3	1.1	11	0.99	0.051172	0.71	0.281	0.76	0.039833	0.13	0.402	249	16	251.5	1.7	251.79	0.33
4	1.2	18	1.1	0.051413	0.50	0.2824	0.53	0.039835	0.14	0.348	259	12	252.5	1.2	251.81	0.35
5	1.3	130	0.86	0.051287	0.09	0.2816	0.15	0.039826	0.13	0.822	253.8	2.0	251.95	0.35	251.76	0.31
6	1.6	181	0.83	0.0512268	0.032	0.2812	0.13	0.039818	0.13	0.971	251.0	0.7	251.64	0.30	251.70	0.31
7	1.3	79	0.70	0.051260	0.13	0.2813	0.18	0.039795	0.13	0.719	252.5	2.9	251.66	0.41	251.56	0.32
8	1.6	38	0.94	0.050936	0.58	0.2794	0.59	0.039789	0.17	0.210	238	13	250.2	1.3	251.52	0.43
9	1.2	34	0.89	0.051398	0.23	0.2819	0.27	0.039772	0.12	0.524	258.7	5.4	252.13	0.61	251.42	0.31
10	1.5	23	0.64	0.051499	0.79	0.282	0.84	0.039770	0.19	0.372	263	18	252.6	1.9	251.40	0.46
11	1.0	22	0.67	0.051355	0.44	0.2815	0.48	0.039757	0.16	0.374	257	10	251.9	1.1	251.32	0.39

(a) 1, 2 etc. are labels for single zircon grains or fragments annealed and chemically abraded[48].

(b) Model Th/U ratio iteratively calculated from the radiogenic 208Pb/206Pb ratio and 206Pb/238U age.

(c) Pb* and Pbc represent radiogenic and common Pb, respectively.

(d) Measured ratio corrected for spike and fractionation only. U fractionation estimated at 0.07 +/- 0.02 %/a.m.u. for FAR analyses, based on repeated analysis of U500.

(e) Corrected for fractionation, spike, and common Pb; common Pb was assumed to be procedural blank: 206Pb/204Pb = 18.11 ± 0.31%; 207Pb/204Pb = 15.28 ± 0.19%; 208Pb/204Pb = 36.80 ± 0.32% (all uncertainties 1-sigma).

(f) Errors are 2-sigma, propagated using the algorithms of 50.

(g) Calculations are based on the decay constants of 49. 206Pb/238U and 207Pb/206Pb ages corrected for initial disequilibrium in 230Th/238U using Th/U [magma] = 3.

A: Geological setting

The Taimyr Peninsula is located in Artic Russia and borders the Kara and Laptev Seas to the NW and NE, respectively (Fig. 1 of the main text). To the south it is bounded by the Yenisei-Khatanga trough, a fault-bounded basin with a thick Mesozoic sedimentary sequence overlying the Permian-to Triassic sedimentary and volcanic rocks[1-3]. The trough separates Taimyr from the Siberian platform hosting the main volumes of Siberian Trap volcanics.

The Taimyr fold belt (Fig. 1) has been divided into three NE-SW-trending structural domains[4], a northern, central and southern separated by major faults[6-10]. The northern domain comprises greenschist facies metamorphosed Neoproterozoic to Early Paleozoic turbidites intruded by granitic plutons of Carboniferous to Permian age, including deformed c.305 granites, which are considered as syn-collisional and undeformed c.265 Ma granites, which are regarded as post-collisional[4,10-13]. The central domain, the Central Taimyr accretionary belt[11] is mainly composed of Neoproterozoic sedimentary and volcanic rocks, including ophiolites, island arc magmatic suites, and continental crust[6,14-19]. These Precambrian rocks were folded and thrust together in the latest Neoproterozoic and unconformably overlain by Vendian-Paleozoic successions[7,8,20]. The southern domain is comprised by non-metamorphosed Ordovician-Permian shallow marine platform (carbonate-dominated) successions covered by Late Permian to Early Triassic sandstones[8,21-25]. It also contains numerous alkaline mafic to felsic intrusive complexes in the form of layered intrusives, plutons, sills and dykes[26-30]. All rocks of the Southern Domain are folded and thrust southward, with a significant decrease in the intensity of both folding and faulting to the [south2,7,8,24,31,32]. In the southern domain, intrusives previously dated to Lower and Middle Triassic cut the Upper Paleozoic silisiclastics and Permo-Triassic lavas (the Taimyr traps) that directly overlie Permian coal-bearing strata[24]. In the southwestern and south-central part of the Taimyr Peninsula the Upper Carboniferous to late Permian succession comprises >1000 m of siliciclastic, coal-bearing sedimentary rocks[25] (Fig. 1). The lower part of this sequence consists of sandstones, siltstones and argilites, with interbedded clayey limestones and coals whereas the upper part of the Permian sequence comprise sandstones and siltstones with interbedded coal layers. The coal layers grow more abundant and thicker towards the west[25].

A complete Permian succession is believed to be present throughout the Yenisei-Khatanga basin[2], and a thickness of the Carboniferous-Permian section here on the order of ca. 7 km has been inferred from seismic data[1,33]. The Carboniferous to Permian sediments of

South Taimyr and the Yenisei-Khatanga Basin were deposited proximally in the foreland deep to the developing Taimyr orogeny grading into the more distal deposits of the Siberian Platform[2].

The volcanic sequence overlying the late Permian terrigenous sedimentary rocks in south Taimyr has been interpreted as the northern continuation of the Siberian traps and are located in the south of the Taimyr Peninsula (in the Southern domain) in a band 50–150 km wide and 1000–1100 km long from the Yenisei Bay (Kara Sea) in the west to Khatanga Bay Laptev seas in the east (Fig. 1). Flood basalts are widespread within the Byranga Mountains of the Taimyr. Here, trachybasalt flows lie directly on the Permian coal-bearing sediments. Above the trachybasalts, andesitic, tholeiitic and picritic basalts, trachyandesites and tephrites[20,21,31,34,35] make up a volcanic succession of 2-3 km total thickness, comparable to the thickness present e.g. in the Norilsk area[36] (>3.5 km) and what can be inferred for the Yenisei-Khatanga depression[33]. There are also doleritic sills and dikes, as well several large layered intrusive complexes cutting both the Permian sedimentary rocks and the lowest part of the effusive sequence present. Most of the mafic intrusive rocks of the Taimyr traps have been found in the southern part of Central Taimyr (Fig.1). They vary from a few meters to more than 1000 m thickness and range from undifferentiated dolerites to alkaline layered intrusions with compositions ranging from peridotitic to gabbroic and dioritic, syenitic and granitic[22,23].

Based on volcano-stratigraphic similarities between volcanogenic rocks in Taimyr and the Norilsk regions, the mafic igneous rocks of the Taimyr Peninsula have been attributed to the Late Permian to Early Triassic activity of the Siberian Traps[31,34,35]. It has, furthermore, been shown that the basalt sequences and dolerites from Taimyr have isotope-geochemical signatures overlapping with those of basalts and dolerites from the main sequences in the Tunguska basin, supporting the link between igneous activity in Taimyr and the Siberian Traps[37]. Recent $^{40}Ar/^{39}Ar$ dating of basalts and one dolerite from Taimyr gives ages overlapping with or slightly younger than ages from the main Siberian Trap sequence in the Tunguska Basin[37,38]. However, some dolerites have also yielded $^{40}Ar/^{39}Ar$-ages of ca. 229-227 Ma[39], significantly younger than the Siberian Traps, leaving the question open as to whether all the mafic intrusives in southern Taimyr are related to the Siberian LIP and if they were emplaced synchronously with the main magmatic pulse recorded in the Tunguska basin. On the western Taimyr Peninsula and on nearby islands alkaline felsic plutonic bodies (syenites, monzonites and (sub)alkaline granites) that intrude the late Carboniferous and Permian sedimentary rocks as well as basalts and dolerites have been dated to ca. 250 - 240

Ma[30], and two ore-bearing alkaline ultramafic-felsic layered intrusives, the Binyuda and Dumtalei intrusions, were suggested to have formed within the same age range, or slightly older, based on rather scattered U-Pb zircon ion probe data[40,41]. These chemically more evolved intrusives have tentatively been interpreted to represent the tailing end of magmatic activity related to the Siberian superplume[30].

Ultramafic to felsic alkaline magmatism in the Siberian Platform is notable but not restricted to the Taimyr area. Alkaline rocks attributed to the Siberian Traps crop out in the Maymecha-Kotuy area, at the NE margin of the Siberian Platform[42,43]. Here, alkaline-ultramafic lavas with rare felsic interlayered flows, dykes and carbonatite bodies constitute the Maymecha-Kotuy alkaline complex, which besides the effusive products includes ca. 30 separate intrusive bodies, such as Guli, a large ultramafic-alkaline intrusion[44,45], considered co-magmatic with some of the alkaline lavas[46].

B: The Dumtalei and Dikarabigai layered intrusive complexes

We have studied the Dumtalei (well TP-43) and the Dikarabigai (TP-42 and TP-55 wells) layered intrusive complexes, located in the southern part of the Taimyr Peninsula, bordering the Yenisei-Khatanga trough (Figs. 1 and 2 of the main text). These layered intrusive complexes are two of at least six known large layered, tabular intrusive complexes in southern Taimyr.

The Dumtalei layered intrusion was intersected by a range of boreholes drilled at the right bank of the Dumtalei River that is the left tributary of the Verkhnyaya (Upper) Taimyra River[29]. The samples studied here were taken from borehole TP-43 (Fig. 2). The intrusion measures approximately 200 km^2 (54 km long and 3-4 km wide) with a thickness of up to 640 m. The magmatic body cuts Carboniferous to Permian mainly terrigenous coal-bearing strata and basalts of the lower part of the Permian-Triassic lava pile showing that the intrusion was emplaced after the first lava flows erupted at the surface. The intrusion is surrounded by a contact aureole of altered volcanic and terrigenous rocks with thicknesses of 90-230 m below the intrusion and 350-540 m above the intrusion[47]. The volcanic rocks in the aureole are altered to fine-grained amphibole-clinopyroxene-plagioclase metasomatic rocks with inclusions of miarolitic pegmatites located in zones of altered amygdaloid basalts.

Four major horizons were recognized within the Dumtalei intrusion. From top to bottom they are comprised by: i) syenite-diorite, ii) sub-alkaline ferrogabbro, iii) sub-alkaline and alkaline troctolite-picrite, dolerites and taxitic peridotites, and iv) contact

gabbrodolerites[29,47]. The syenite-dioritic horizon, which is dated here (Fig. 3d of the main text), forms a solid interval at the roof of the intrusion. It is composed of quartz-bearing and quartz-free syenites, amphibole- and pyroxene-bearing syenites, monzonites, monzodiorites and diorites. Rocks are comprised of K-feldspars (50-60%), albite-oligoclase (20-25%), and quartz (0-15%). Amphibole, mica, apatite, titanite, zircon, magnetite and sulfides form the minor (5% in total) constituent of rock. The thickness of this horizon is 100 m. The syenite-diorites occurring at the top of the Dumtalei layered intrusion have a sharp non-quenched contact with the underlying titanomagnetite-bearing sub-alkaline ferrogabbro. Syenite and diorite-pegmatite schlieren occur in the ferrogabbro at the contact with the syenite horizon. Apophyses of syenite are cutting into the overlying country rocks. The syenites in the Dumtalei intrusion have been interpreted to result from magma-unmixing of an initial trachybasaltic parent melt to sialic and mafic liquids[23]. The ferrogabbroic portions of the layered intrusive has initial isotope ratios of: $^{87}Sr/^{86}Sr_{250Ma}$ = 0.70454-0.70494 and $^{143}Nd/^{144}Nd_{250Ma}$ = 0.51250-51257[41].

The Dikarabigai intrusive complex from which two drill cores (TP-42 and TP-55) were studied here, form stocks, sheeted bodies and dikes of sub-alkaline and alkaline rocks (Fig. 2). It is up to ca. 900 m thick (borehole TP-42; borehole TP-55 only reached 500 m down into the intrusion) and cuts Upper Permian terrigeneous and coal-bearing sedimentary rocks as well as, in places, the lower part of the Permo-Triassic lava pile (borehole TP-55). The areal extent of the intrusion is not constrained. The intrusion has a more than 600 m thick contact aureole into the sedimentary rocks and overlying lowest volcanic rocks as observed from the upper contact of the intrusion in borehole TP-42.

Three major phases of the Dikarabigai intrusive complex are recognized: i) sub-alkaline gabbro and monzodiorite, ii) syenite and iii) quartz syenite and monzonite. Gabbro and monzodiorite occur as sheeted bodies and dikes while layered intrusions consisting of monzodiorite, monzonite and syenite comprise the main intrusion drilled in the TP-42 borehole (Fig. 2). The intrusion is ca. 900 m thick and cuts Upper Permian terrigeneous and coal-bearing sedimentary rocks. Five trachydoleritic dikes of 0.1 to 1.2 m thickness cut the layered intrusion. A > 600 m thick contact aureole above the intrusion can be observed in the overlying sedimentary rocks and lowest volcanic rocks. This is expressed by alkaline-silica metasomatic rocks with calcite mineralization and as epidote-pyroxene-garnet-calcite skarns with anhydrite, quartz, scapolite and mica. Associated with the skarn zones there are also disseminated magnetite and pyrrhotite mineralization forming massive chalcopyrite-

pyrrhotite veins with up to 15 cm thickness. The samples studied here are syenitic (TP-42-1) to monzosyenitic (TP-42-2), where the latter appears to be a coarser grained differntiate of the former. The contact between the two is gradual and the coarse grained monzosyenitc appears as enclosed pockets in the syenite. The syenitic phase is coarse grained and in addition to the dominant K-feldspar contents and plagioclase, contains amphibole as the main mafic phase (ca. 5 %). There are a few dispersed pyroxenes and abundant titanite and oxides (ca. 1 % each). Accessory apatite is also present. The monzosyenitic phase apart from being dominated by large K-feldspars, contain up to 20 % plagioclase, ca. 5 % amphibole and ca. 2 % titanite. A few pyroxene crystals, as well as about 0.5 % oxides and very minor magmatic calcite are also present. Apatite occurs as an accessory mineral. In borehole TP-55 only the upper 500 m of the layered intrusion was cored. Here it consists of interbedded syenites and monzonites and cuts alkaline basalts of the lava pile showing that also the Dikarabigai intrusive complex was emplaced after the first lava flows at the surface. The syenitic sample studied here contains ca. 15 % plagioclase, more than 5 % biotite, 1-2 % oxides and a few dispersed amphibole grains in addition to the dominant K-feldspar. Titanite is an abundant accessory mineral, and there is also some accessory apatite and a couple of magmatic calcite grains present.

References

1. *Kushnir, D.G. Paleozoic swells in the north of central and western Siberia.* Geotectonics ***40(5)****, 399-404 (2006).*
2. *Afanasenkov, A.P., et al. The tectonics and stages of the geological history of the Yenisei–Khatanga Basin and the conjugate Taimyr Orogen.* Geotectonics ***50(2)****, 161-178 (2016).*
3. *Vernikovsky V.,* Shemin G., Deev E, Metelkin D., Matushkin N. & Pervukhina N. Geodynamics and Oil and Gas Potential of the Yenisei-Khatanga Basin (Polar Siberia). *Minerals* **8**, 510 (*2018).* doi: 10.3390/min8110510
4. Zhang, X., Pease, V., Carter, A., Kostuychenko, S., Suleymanov, A. & Scott, R. Timing of exhumation and deformation across the Taimyr fold–thrust belt: insights from apatite fission track dating and balanced cross-sections. *Geological Society, London, Special Publications* **460(1)**, 315-333 (2018).
5. Zonenshain, L.P., Kuzmin, M.I. & Natapov, L.M. Geology of the USSR: A Plate Tectonic Synthesis. (ed. Page, B.M.). Geodyn. Ser. **21**. AGU, Washington, D.C. (1990)
6. Urvantsev, N.N. Taimyr folded zone. *Bull. Norilsk Kombinat* **4-12** (1949).

7. Pogrebitsky, Y.E. Paleotectonic Analysis of the Taimyr Fold System. Nedra, Leningrad, 248 pp. (in Russian; 1971).

8. Bezzubtsev, V.V., Zalyaleyev, R. & Sakovich, A. Geological map of mountainous Taimyr 1: 500 000: explanatory notes. Krasnoyarskgeologia, Krasnoyarsk (in Russian; 1986).

9. Uflyand, A., Natapov, L., Lopatin, V. & Chernov, D. On the Taimyr tectonic nature. *Geotectonics* **6**, 76-79 (1991).

10. Vernikovsky, V.A., Neimark, L.A., Ponomarchuk, V.A. & Vernikovskaya, A.E. Geochemistry and age of collisional granitoides and metamorphites of the Kata microcontinent (Northern Taimyr). *Russian Geology and Geophysics* **36(12)**, 50–64 (1995).

11. Vernikovsky, V.A. & Vernikovskaya, A.E. Central Taimyr accretionary belt (Arctic Asia): Meso–Neoproterozoic tectonic evolution and Rodinia breakup. Precambrian Research ***110(1-4)****, 127-141 (2001).*

12. Pease, V. & Scott, R.A. Crustal affinities in the Arctic Uralides, northern Russia: significance of detrital zircon ages from Neoproterozoic and Palaeozoic sediments in Novaya Zemlya and Taimyr. Journal of the Geological Society ***166(3)****, 517-527 (2009).*

13. Pease, V. L., Kuzmichev, A. B. & Danukalova, M. K. The New Siberian Islands and evidence for the continuation of the Uralides, Arctic Russia. *Journal of the Geological Society* **172(1)**, 1-4 (2015).

14. Vernikovsky, V.A., Vernikovskaya, A.E., Chernykh, A.I. & Mel'gunov M.S. Petrology and geochemistry of Taimyr Riphean ophiolites. *Russian Geology and Geophysics* **37(1)**, 103–121 (1996).

15. Vernikovsky, V.A. Taimyr fold area: evolution of the earth crust and the main problems of tectonics. In III International Conference on Arctic Margins-ICAM III, Celle (Germany), 12-16 October 1998: Abstracts, 196-197, (1998).

16. Vernikovsky, V.A., Metelkin, D.V., Vernikovskaya, A.E., Sal'nikova, E.B., Kovach, V.P. & Kotov, A.B. The oldest island arc complex of Taimyr: Concerning the issue of the Central-Taimyr accretionary belt formation and paleogeodynamic reconstructions in the Arctic. *Dokl. Earth Sci.* **436(2)**, 186–192 (2011).

17. Khain, V.E., Gusev, G.S., Khain, E.V., Vernikovsky, V.A. & Volobuyev, M.I. Circum-Siberian Neoproterozoic Ophiolite Belt. *Ofioliti* **22(2)**, 195–200 (1997).

18. Pease, V., Gee, D.G., Vernikovsky, V., Vernikovskaya, A. & Kireev, S. Geochronological evidence for late-Grenvillian magmatic and metamorphic events in central Taimyr, northern Siberia. *Terra Nova* **13(4)**, 270-280 (2001).

19. *Priyatkina, N., et al. The Proterozoic evolution of northern Siberian Craton margin: a comparison of U–Pb–Hf signatures from sedimentary units of the Taimyr orogenic belt and the Siberian platform.* International Geology Review ***59(13)****, 1632-1656 (2017). DOI: 10.1080/00206814.2017.1289341*
20. Proskurnin V.F., et al. Rhyolite–granite association in the Central Taimyr zone: evidence of accretionary-collisional events in the Neoproterozoic. *Russian Geology and Geophysics* **55**, 18–32 (2014).
21. *Zolotukhin V.V., et al. Magnesian basites of west of the Siberian platform and questions of nickel-plating. (Ed Sobolev V.S.),* Novosibirsk: Science, *225pp. (In Russian; 1984).*
22. *Nagaitseva N.N., Ermolaev I.K. & Kolesova N.Y. Nickel-bearing intrusive complexes of the central part of the Mountain Taimyr// Rudnomagmatic complexes of the northwest of the Siberian platform and Taimyr - L.: Sevmorgeologiya, 95-112 (In Russian; 1985).*
23. *Ryabov V.V. Liquefaction in natural glasses (on the example of traps).* Novosibirsk: Science, *223 pp. (1989).*
24. *Inger, S., Scott, R.A. & Golionko, B.G. Tectonic evolution of the Taimyr Peninsula, northern Russia: implications for Arctic continental assembly.* Journal of the Geological Society ***156(6)****, 1069-1072 (1999).*
25. *Ershova, V.B., Prokopiev, A.V. & Khudoley, A.K. Devonian–Permian sedimentary basins and paleogeography of the Eastern Russian Arctic: an overview.* Tectonophysics ***691****, 234-255 (2016).*
26. Ravich M.G. & Chaika L.A. Differentiated intrusion of trap formation from Taimyr folded area. Izv. AN SSSR, ser. geol **1**, 50-64 (in Russian; 1956).
27. Zolotukhin, V.V. Features of Tulay-Kiryaka differentiated intrusion from Taimyr: Nauka, Novosibirsk, 111 pp. (in Russian; 1990).
28. *Al'mukhamedov, A.I., Zolotukhin, V.V., Al'mukhamedov, E.A., Sandimirova, G.P. & Pakhol'chenko, Y.A. Geochemical model of basalt magma hybridisation exemplified by the Tulai-Kiryaka intrusion (Taimyr).* Russian Geology and Geophysics ***4****, 42-50 (1993).*
29. Komarova, M.Z., Kozyrev, S.M., Kokorin, N.I., and Knauf, V.V. Layered intrusion of the Dyumtaley River: petrology, ore content. In: (eds. Simonov, O.N., Malich, N.S.) Bowels of Taimyr. Norilsk, *Taimyrgeolcom*, 42-67, Simonov (1999).
30. *Vernikovsky, V.A., Pease, V.L., Vernikovskaya, A.E., Romanov, A.P., Gee, D.G. & Travin, A.V. First report of early Triassic A-type granite and syenite intrusions from Taimyr: product of the northern Eurasian superplume?.* Lithos ***66(1-2)****, 23-36 (2003).*

31. Gurevitch, E., Westphal, M., Daragan-Suchov, J., Feinberg, H., Pozzi, J.P. & Khramov, A.N. Paleomagnetism and magnetostratigraphy of the traps from Western Taimyr (northern Siberia) and the Permo-Triassic crisis. *Earth and Planetary Science Letters* **136(3-4)**, 461-473 (1995).
32. *Khain, V.E. Tectonics of continents and oceans (year 2000). Moscow, Scientific World, 606 pp. (2001).*
33. *Ershova, V.B., et al. Trans-Siberian Permian rivers: A key to understanding Arctic sedimentary provenance.* Tectonophysics ***691****, 220-233 (2016).*
34. *Salmanov, A.P. Basaltic komatieits of the southwestern Taimyr / / Izv. AN SSSR. Ser. geol.* ***11****, 132-136 (In Russian; 1987).*
35. Zolotukhin, V.V. & Al'mukhamedov A.I. Traps of the Siberian platform, In, (ed. J.D.Macdougall) Continental Flood Basalts. Norwell, Mass., 273-310 (1988).
36. *Lind, E.N., Kropotov, S.V., Czamanske, G.K., Gromme, S.C. & Fedorenko, V.A. Paleomagnetism of the Siberian flood basalts of the Noril'sk area: a constraint on eruption duration.* International Geology Review ***36(12)****, 1139-1150 (1994).*
37. *Reichow, M.K., et al. Petrogenesis and timing of mafic magmatism, South Taimyr, Arctic Siberia: A northerly continuation of the Siberian Traps?.* Lithos ***248****, 382-401 (2016).*
38. *Reichow, M.K., et al. The timing and extent of the eruption of the Siberian Traps large igneous province: Implications for the end-Permian environmental crisis.* Earth and Planetary Science Letters ***277(1-2)****, 9-20 (2009).*
39. *Walderhaug, H.J., Eide, E.A., Scott, R.A., Inger, S. & Golionko, E.G. Palaeomagnetism and 40Ar/39Ar geochronology from the South Taimyr igneous complex, Arctic Russia: a Middle-Late Triassic magmatic pulse after Siberian flood-basalt volcanism.* Geophysical Journal International ***163(2)****, 501-517 (2005).*
40. *Badanina, I.Y., Malitch, K.N. & Romanov, A.P. September. Isotopic-geochemical characteristics of the ore-bearing ultramafic-mafic intrusions of western Taimyr, Russia.* Doklady Earth Sciences ***458(1)****, 1165-1167 (2014).*
41. *Malitch, K.N., Badanina, I.Y., Romanov, A.P. & Slusjenkin, S.F. U-Pb age and Hf-Nd-Sr-Cu-S isotope systematics of the Binyuda and Dyumtaley ore-bearing intrusions (Taimyr, Russia).* Lithosphere ***1****, 107-128 (in Russian; 2016).*
42. *Arndt, N., Chauvel, C., Czamanske, G. & Fedorenko, V. Two mantle sources, two plumbing systems: tholeiitic and alkaline magmatism of the Maymecha River basin, Siberian flood volcanic province.* Contributions to Mineralogy and Petrology ***133(3)****, 297-313 (1998).*

43. *Sobolev, A.V., Sobolev, S.V., Kuzmin, D.V., Malitch, K.N. & Petrunin, A.G. Siberian meimechites: origin and relation to flood basalts and kimberlites.* Russian Geology and Geophysics ***50(12)****, 999-1033 (2009).*

44. Egorov, L.S. Ijolite– Carbonatite Plutonism (by Example of the Maimecha–Kotui Complex of Polar Siberia), Leningrad: Nedra (in Russian; 1991).

45. *Kogarko, L.N. & Zartman, R.E. A Pb isotope investigation of the Guli massif, Maymecha-Kotuy alkaline-ultramafic complex, Siberian flood basalt province, Polar Siberia.* Mineralogy and petrology ***89(1-2)****, 113-132 (2007).*

46. *Fedorenko, V. & Czamanske, G. Results of new field and geochemical studies of the volcanic and intrusive rocks of the Maymecha-Kotuy area, Siberian flood-basalt province, Russia.* International Geology Review ***39(6)****, 479-531 (1997).*

47. *Sluzhenikin S. F., et al. Platinum bearing capacity of the Dumtalei hyperbasite-basite layered titaniferous massif in the Central Taimyr. / Platinum of Russia. M.* Geoinformmark JSC, *107-123 (1999).*

48. *Mattinson, J.M. Zircon U–Pb chemical abrasion ("CA-TIMS") method: combined annealing and multi-step partial dissolution analysis for improved precision and accuracy of zircon ages.* Chemical Geology ***220(1-2)****, 47-66 (2005).*

49. *Jaffey, A.H., Flynn, K.F., Glendenin, L.E., Bentley, W.T. & Essling, A.M. Precision measurement of half-lives and specific activities of U 235 and U 238.* Physical Review C ***4(5)****, pp.1889 (1971).*

50. *Schmitz, M.D. & Schoene, B. Derivation of isotope ratios, errors, and error correlations for U-Pb geochronology using* ^{205}Pb*-*^{235}U*-(*^{233}U*)-spiked isotope dilution thermal ionization mass spectrometric data.* Geochemistry, Geophysics, Geosystems ***8(8)****, (2007).* **https://doi.org/10.1029/2006GC001492**

51. Middlemost, E.A. Naming materials in the magma/igneous rock system. *Earth-Science Reviews* **37(3-4)**, 215-224 (1994).
